# Microbiological Evaluation of Water Quality from Urban Watersheds for Domestic Water Supply Improvement 

**DOI:** 10.3390/ijerph8124460

**Published:** 2011-11-30

**Authors:** A. Mark Ibekwe, Shelton E. Murinda, Alexandria K. Graves

**Affiliations:** 1 U.S. Salinity Laboratory, USDA-ARS, 450 West Big Springs Road, Riverside, CA 92507, USA; 2 Department of Animal and Veterinary Sciences, California State Polytechnic University Pomona, 3801 West Temple Avenue, Pomona, CA 91768, USA; Email: semurinda@csupomona.edu; 3 Department of Soil Sciences, North Carolina State University, Williams Hall 3411E, P.O. Box 7619, Raleigh, NC 27695, USA; Email: alexandria_graves@ncsu.edu

**Keywords:** pathogenic *Escherichia coli*, indicator bacteria, surface water, sediment, contamination, watershed

## Abstract

Agricultural and urban runoffs may be major sources of pollution of water bodies and major sources of bacteria affecting the quality of drinking water. Of the different pathways by which bacterial pathogens can enter drinking water, this one has received little attention to date; that is, because soils are often considered to be near perfect filters for the transport of bacterial pathogens through the subsoil to groundwater. The goals of this study were to determine the distribution, diversity, and antimicrobial resistance of pathogenic *Escherichia coli* isolates from low flowing river water and sediment with inputs from different sources before water is discharged into ground water and to compare microbial contamination in water and sediment at different sampling sites. Water and sediment samples were collected from 19 locations throughout the watershed for the isolation of pathogenic *E. coli*. Heterotrophic plate counts and *E. coli* were also determined after running tertiary treated water through two tanks containing aquifer sand material. Presumptive pathogenic *E. coli* isolates were obtained and characterized for virulent factors and antimicrobial resistance. None of the isolates was confirmed as Shiga toxin *E. coli* (STEC), but as others, such as enterotoxigenic *E. coli* (ETEC). Pulsed field gel electrophoresis (PFGE) was used to show the diversity *E. coli* populations from different sources throughout the watershed. Seventy six percent of the isolates from urban sources exhibited resistance to more than one antimicrobial agent. A subsequent filtration experiment after water has gone through filtration tanks containing aquifer sand material showed that there was a 1 to 2 log reduction in *E. coli* in aquifer sand tank. Our data showed multiple strains of *E. coli* without virulence attributes, but with high distribution of resistant phenotypes. Therefore, the occurrence of *E. coli* with multiple resistances in the environment is a matter of great concern due to possible transfer of resistant genes from nonpathogenic to pathogenic strains that may result in increased duration and severity of morbidity.

## 1. Introduction

The Santa Ana River (SAR) is the largest river in the Santa Ana Region of southern California and it is a major source of domestic water supply for over 2 million people that live in Orange County, California. The Santa Ana River is critical for replenishment of Orange County’s Groundwater Basin since over 2 million residents in Orange County depend on groundwater for 75% of their water supply. Any factor in the watershed which degrades the river affects the drinking water supply. The river extends from its headwaters in the San Bernardino Mountains into the Prado Basin and Santa Ana Canyon. Below Prado Dam, there are extensive facilities to recharge much of the flows in the River into the underlying groundwater basin. Sources of non-point contaminants into the river may be from municipal wastewater, agricultural waste discharges, urban runoffs, and a combination of the above factors. Currently, the Santa Ana River in southern Californian is impacted by one of the highest concentrations of cattle in the United States. The watershed is undergoing drastic changes. In general, the varying land uses in the middle SAR (Chino Basin) watershed include agriculture, open space, and rapidly growing urban areas [1]. In 1995, approximately 340 animal-confinement facilities having over 386,000 animals, mostly dairy cows, operated within the area that is mostly drained by Chino, Cypress, and Cucamonga Creeks. Pollutants in the watershed mainly consist of pathogens and nutrients due to the densely populated areas, agricultural activities, and urban and storm-water runoff in the region. Different federal, state, and private agencies have monitored fecal bacterial composition in the surface water [1,2], but little has been done to determine the different *Escherichia coli* strains within the water bodies. 

*E. coli* are very diverse in the environment with about 173O, 103K, and 56H antigen and the numbers of newly discovered antigens is increasing [3]. Most *E. coli* are nonpathogenic, but there are some such as *E. coli* O157:H7 that cause human diseases such as hemorrhagic colitis (HC) and hemolytic uremic syndrome (HUS). In addition to *E. coli* O157:H7, there are other *E. coli* pathogroups that causes diseases in human such as enteropathogenic *E. coli* which causes diarrhea in children especially in developing countries, enterotoxigenic *E. coli* which causes traveler’s diarrhea and others [3]. There is an extensive review of sources of pathogenic *E. coli* in the environment [4], but their distributions in urban water has been limited to very few studies [5,6]. Due to the increasing urbanization and the large number of cattle in the studied watersheds, the health risk from pathogenic *E. coli* is a major concern to drinking water quality. There is virtually no information on the occurrence of pathogenic *E. coli* in the middle Santa Ana River watershed despite the high concentration of cattle in the watershed. 

Most pathogenic *E. coli* are commonly carried by healthy cattle in their feces. The fecal excretion of these organisms by cattle appears to be seasonal, with excretion rates highest in spring and late summer [7,8]. This studies sought to characterize pathogenic *E. coli* isolates obtained in terms of their virulence profiles, and pulsed field gel electrophoresis (PFGE) genomic profiles. PFGE DNA banding patterns, in conjunction with virulent factors, may assist in the epidemiologic tracing of pathogenic *E. coli* isolates of medical concern. The goals of this study were to determine the distribution, diversity, and antimicrobial activities of pathogenic *E. coli* isolates from low flowing river water and sediment with inputs from different sources before water is discharged into ground water and to compare microbial contamination in water and sediment at different sampling sites. We also incorporated the evaluation of fecal bacterial contamination of drinking water aquifer sand material at a specific site that receives water from the above sources before discharge into ground water.

## 2. Materials and Methods

### 2.1. Study Area and Sample Collection

**Figure 1:**
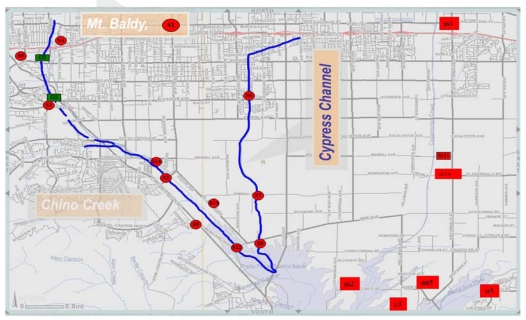
Sites used for the study along Chino Basin also known as the middle Santa Ana River Watershed (MSAR). Chino Creek and Cypress channels are the two main channels in the basin with inputs from urban and agricultural activities, respectively. Both Creeks empty into the Santa Ana River.

The study area is as previously described [9], which outlines the sampling points and their relative locations (Figure 1; Table 1). Surface water samples and sediment were collected from a natural/open-space location (S1, M1) to evaluate bacterial contributions from natural or background source. The S1 sampling point is located in National Forest land. Effluent from three wastewater treatment plants (S11WW, S13 and S14) were also analyzed (Table 1). All sampling locations and their land use types are listed in Table 1. Reference samples were taken quarterly for 12 months. All samples were collected at the water surface in duplicate in sterile recipients, and sediments from the bank of the river, stored at 4 °C, and analyzed within 6–8 hours. Sediment samples were collected in duplicate from the river banks with a stainless steel instrument and analyzed within 24 hours. 

The topography in the Santa Ana River watershed ranges from steep, rugged mountains with peaks as high as 3,261 m above sea level, to a broad alluvial-filled valley, bordered by the San Gabriel/San Bernardino Mountains to the northeast and the elevated Perris Block/San Jacinto Mountains to the south (Figure 1). The Santa Ana River is the main tributary draining the valley. The Chino Basin study area, located in the northwestern part of this watershed, was formed as a result of tectonic activity along major fault zones [10]. The bottom of the basin—the effective base of the freshwater aquifer—consists of relatively impermeable sedimentary and igneous bedrock formations that are exposed at the surface in the surrounding mountains and hills. Sediments eroded from the surrounding mountains have filled the Chino Basin, providing reservoirs for ground water. In the deepest portions of the basin, these sediments are greater than 304 m thick. The sediments consist of geologically old and young alluvium. The thickness of the older alluvium varies from about 60 m near the southwestern end of the Chino Basin to over 334 m in the eastern parts of the valley and averages about 152 m throughout the basin. 

**Table 1:** Sampling locations for MSAR pathogen source evaluation study. *

Site #	Site locations	Land use	Geographic positioning system (GPS)
**S1**	Ice House Canyon	Open Space	N34° 15.057 min.; W117° 37.977 min; 1,447 m elevation
M1	Cucamonga Creek. at OCWD Ponds	Open Space	San Bernardino County Flood Control District (SBCFCD)
**S2**	Chino Creek at Central Ave.	Urban runoff	N33° 58.420 min.; W117° 41.302 min; 174 m elevation
**S3**	Chino Creek at Schaefer Ave.	Urban runoff	N34° 0.246 min.; W117° 43.628 min; 207 m elevation
**S4**	San Antonio Wash at County Drive	Urban runoff + Commercial wash out	N30° 1.543 min.; W117° 43.652 min; 222 m elevation;
**S5**	Chino Creek. at Riverside Drive	Urban runoff	N34° 1.144 min.; W117° 44.204 min; 207 m elevation;
**S6**	Cypress Channel at Schaefer Ave.	Agricultural Runoff	N34° 0.262 min.; W117° 39.766 min 208 m elevation;
**S7**	Cypress Channel at Kimball Ave.	Agricultural Runoff	N33° 58.113 min.; W117° 39.624 min 177 m elevation;
**S8**	Cypress Channel at Golf Course	Agricultural Runoff	N33° 57.057 min.; W117° 39.555 min; 160 m elevation;
**S9**	Big League Dreams at storm drain	Urban runoff	N33° 57.364 min.; W117 °40.788 min; 163 m elevation;
S11ww	Cucamonga Creek at Regional Water Recycling Plant #1	Effluent from wastewater treatment plant	N34°; 1.853 min; W117° 35.946 min; Altitude:246 m
S11ur	Cucamonga Creek at Regional Water Recycling Plant #1	Urban runoff+ wastewater	N34°; 1.853 min; W117° 35.946min; Altitude:246 m.
**S12**	Chino Creek at Pine Ave.	Urban runoff+ wastewater	N33° 56.941 min.;W117° 39.986 min;155 m elevation;
**S13**	Inland Empire Utilities Agency (IEUA) Regional Water Recycling Plant #5	Effluent from wastewater treatment plant	N33° 57.840 min.; W117 ° 40.826 min; 180 m elevation;
**S14**	IEUA Carbon Canyon Waste Reclamation Facility (CCWRF)	Effluent from wastewater treatment plant	N33 ° 58.799 min.; W117° 41.655 min; 184 m elevation;
ST2	Santa Ana River at Prado Dam	Urban Runoff	N33°; 54.737 min; W117° 38.711 min Altitude: 141 m.
C3	Prado Park outlet	Urban Runoff+ waste water discharge	N33°; 56.402 min; W117° 38.763min, 166 m
ST5	Santa Ana River at River road	Urban Runoff	N33°; 55.405 min; W117° 35.894 min Altitude:155 m.
M5	OCWD (Prado)Wetlands Effluent	Wetland treated (bacteria loaded) Orange County Water District (OCWD	N33°; 54.737 min; W117° 38.711 min Altitude: 141 m.

* From Ibekwe *et al*. [9] with slight changes.

The surface outcrop is commonly distinguishable by its red-brown or brick-red color and is generally more weathered than the overlying younger alluvium. The younger alluvium occupies streambeds, washes, and other areas having recent sedimentation. The thickness of the younger alluvium varies from over 30 m near the mountains to just a few meters in the center of the valley. The younger alluvium generally covers most of the northern half of the Chino Basin in undisturbed areas [10].

The stratigraphy of the Chino Basin can be described by two natural divisions: (1) the pervious formations that comprise the ground-water reservoirs are termed the water-bearing sediments and (2) the less pervious formations that enclose the ground-water reservoirs are termed the consolidated bedrock. The consolidated bedrock is further differentiated as metamorphic and igneous rocks of the basement complex partially overlain by consolidated sedimentary rocks. The water-bearing sediments overlie the consolidated bedrock, with the bedrock formations coming to the surface in the surrounding hills and highlands. 

Most recharge to the ground-water reservoirs of the Chino Basin is from percolation of direct precipitation and infiltration of stream flow within tributaries exiting the surrounding mountains and hills and within the Santa Ana River. Potential sources of recharge in the Chino Basin include the following: (1) infiltration of flow (and, locally, imported water) within unlined stream channels overlying the basin, (2) infiltration of storm water flow and municipal wastewater discharges within the channel of the Santa Ana River, (3) underflow from the saturated sediments and fractures within the bounding mountains and hills, (4) artificial recharge at spreading grounds of storm water, imported water, and recycled water, (5) underflow from seepage across the bounding faults, (6) intermittent underflow from adjacent basins, and (7) deep percolation of precipitation and returns from use [10].

Warm, dry summers and cool, moist winters characterize the climate of the study area. Average annual precipitation ranges from about 450 mm in the lower part of the Chino Basin to about 1,000 mm in the San Gabriel Mountains. Most precipitation occurs during the winter rainy season between November and March. Average precipitation for January ranges from about 76 mm in the southern valley to more than 254 mm in the mountains, whereas average precipitation for July is less than 2.54 mm across the whole basin [11]. The spatial distribution of average monthly precipitation is similar for most months and is characterized by the topographic effect of the San Gabriel Mountains. For example, although average November precipitation has a spatial distribution that is similar to the January pattern, the average January precipitation is over 30% greater than that for all other months. Air temperatures across the basin in the winter are cool, with average daily temperatures in January ranging from about 2 °C in the north, resulting in persistent mountain snowpack at the higher altitudes, to as high as 15 °C in the southern valley. Average daily air temperatures for July can be quite warm, ranging from 18 to 27 °C.

### 2.2. Enumeration of TC, FC, and *E. coli* from Chino Basin

Water samples were processed in the laboratory within six hours of sample collection. All water samples were transported on ice to the laboratory and analyzed by adding 100 mL of water sample to a Colilert vessel and processing following the manufacturer’s protocol. TC, FC, and *E coli* populations were enumerated and expressed as Most Probable Number (MPN/100mL). For isolation of *E. coli* colonies from Colilert vessels, 100 μL liquid sample was removed from positive wells, then spread plated onto Chromagar ECC agar (CHROMagar Microbiology, Paris, France), and was incubated at 37 °C for 24 h. Individual colonies of pure cultures that were isolated were stored at –80 °C for further characterization. Moist sediment samples (10 g) were diluted with 90 mL of phosphate buffered saline (PBS) water (0·0425 g/L KH_2_PO_4_ and 0·4055 g/L MgCl_2_) and shaken for 15 minutes. Ten mL of the suspension was added to Colilert vessel, diluted 1:10 and mixed. One mL from the 1:10 dilution was transferred to another vessel and was further diluted 1:1,000, and an aliquot was added to the Colilert media, mixed, then sealed in QuantiTrays and incubated at 37 °C for 24 h. Samples were processed following the manufacturer’s protocol in accordance with method 9223 [12].

### 2.3. Isolation of Pathogenic *E. coli* from Chino Basin

One gram or 1 mL of environmental samples was added to 9 mL of PBS, vortexed briefly, serially diluted and plated for the enumeration of *E. coli* O157 on Harlequin cefixime-tellurite sorbitol MacConkey (CT-SMAC) agar with BCIG (5-bromo-4-chloro-3-indoxyl-β-D-glucuronide) containing 0.05 mg of cefixime L**^−^**^1^ and 2.5 mg of tellurite L**^−^**^1^ (LAB M: IDG–Lancashire, UK). The plates were incubated at 37 °C for *E. coli* O157 for 16 h. Six sorbitol-negative, translucent colonies per sample were tested by multiplex PCR to determine the presence of the three genes. Additionally, isolates that were sorbital positive or β-glucuronidase positive (red/pink colonies with a purple center or green colonies) were enumerated as other *E. coli* or non O157 or presumptive pathogenic *E. coli.* The presumptive pathogenic *E. coli* isolates were probed for the following genes: heat labile toxin (LT), heat stable toxins a & b (STa/STb), shiga-like toxins 1 & 2 (*stx*1/*stx*2), cytotoxin necrotizing factors 1 & 2 (*cnf*1/*cnf*2, intimin (*eaa*), O and H types [13]. These analyses were performed at the *E. coli* Reference Center (The Pennsylvania State University, University Park, PA, USA).

### 2.4. Pulsed-Field Gel Electrophoresis

XbaI PFGE analysis was carried out as previously described [14]. Comparison of digested profiles to identify restriction enzyme digestion pattern clusters (REPCs) was performed with the BioNumerics software, version 5.0 (Applied Maths, Austin, TX, USA). Fingerprints were clustered by using the Jaccard coefficient evaluated by the unweighted-pair group method (UPGMA). A tolerance and optimization of 1% was allowed to account for gel-to-gel differences. Isolates were considered to be related and to belong to the same PFGE cluster if their similarity index was >85%, according to Tenover’s criteria (≤6 bands of difference) [15].

### 2.5. Antimicrobial Resistance

Antimicrobial susceptibility tests (phenotypes) of pathogenic *E. coli* isolates were done using a disk diffusion assay following CLSI standards [16]. Mueller-Hinton II agar (Difco) was used and cells were harvested from the surface of the medium with a cotton swab after 24 h growth at 37 °C. Cells were suspended in sterile saline (0.85% NaCl) and cell density was adjusted to a 0.5 McFarland turbidity standard. The diluted cells were plated, and then antibiotic disks were placed on them. Following incubation (24 h at 37 °C), zone sizes (diameter) were measured to two decimal points and were used for quantitative analysis. Isolates resistant to two or more antimicrobials were defined as multiply drug resistant. *E. coli* ATCC 25922 (American Type Culture Collection, Manassas, VA, USA) was included in each assay as a negative control strain. Antimicrobial agents were tested with BD BBL Sensi-Disc antimicrobial susceptibility test discs (Becton Dickinson & Co., Sparks, MD, USA) with the breakpoints (μg mL**^−^**^1^) indicated as follows: amoxocillin/clavulanic acid, 20/10 μg, ampicillin, 10 μg, cephalothin, 30 μg, erythromycin, 15 μg, rifampin, 5 μg, streptomycin, 10 μg, and tetracycline, 30 μg. 

Multiplex PCR screens were performed on the isolates using genes encoding for ampicillin resistance (*bla*_TEM_), tetracycline resistance (*tet* A, *tet* B, and *tet* C), and streptomycin resistance (*aadA*I). Details of primers, annealing temperatures, and amplicon sizes are as previously provided [9,17,18].

### 2.6. Sampling Collection During Sand Filtration Experiment

Water samples were collected from the Santa Ana River at Orange County Water District Field Station. The water consists of source water (water from the river) and filtrate water (water from aquifer sand material after passing through a sand filtration system).This process was repeated three times to determine reliability of data. The experiment was conducted in a 1.2 × 1.2 × 1.8 m filtration tank built with stainless steel outside the field station. Aquifer sand material was heterogeneous native lake sediment that had been processed through a sand washing plant to remove the majority of silt and clay particles [19,20]. The material was trucked to the station and packed into the tanks. Samples from aquifer material were collected on day 15 at the end of each experiment. Source water was obtained from the Santa Ana River water stored in an artificial lake. The water ran through a 1 inch PVC pipe into the sand filtration unit. The water runs another 1 m through the filtration tank containing aquifer materials and collected at the outlet for analysis of fecal bacteria. 

### 2.7. Enumeration of Heterotrophic Bacteria and *E. coli* in Water Before and After Sand Filtration

Water sample was collected in 1-L sterile bottles, transported on ice to the laboratory, and processed within 6 h using standard procedure [21]. Various dilutions and volumes were filtered with the goal of achieving 30–300 colonies per dilution. Surface water samples was vortexed and volumes of 100, 10 and 1 mL were filtered in phosphate buffered saline (PBS) water (0.0425 g/L KH_2_PO_4_ and 0.4055 g/L MgCl_2_) to obtain the best sample conditions. Tenfold and 100-fold dilutions were also prepared in PBS, vortexed, and 1 mL of each dilution was filtered in duplicate. Volumes of 1 mL, 10 mL and 100 mL (via membrane filtration) were plated onto tryptic soy agar (TSA) (for heterotrophic plate counts [HPC]) and sorbitol-MacConkey agar (SMAC-BCIG without cefixime-tellurite) for *E. coli*, and incubated at 37 °C for 24 h and colonies were enumerated. *E. coli* strains are β-glucuronidase positive and/or sorbitol positive, so produce pink/red colonies with a purple center, or green colonies (some may be translucent with a green center). 

### 2.8. Statistical Analysis

All analyses (PFGE, culture isolation and PCR) were performed in triplicate, and the data shown in the graphs are the average of three separate measurements conducted. Thus, an analysis of variance (ANOVA) was conducted with log_10_-transformed density of *E. coli* bacteria using SAS version 9.1 [22] to determine statistical significant differences using Tukey’s studentized range (HSD) test for mean separation. 

## 3. Results

### 3.1. Fecal Indicator Bacterial Concentrations in Chino Basin

**Figure 2:**
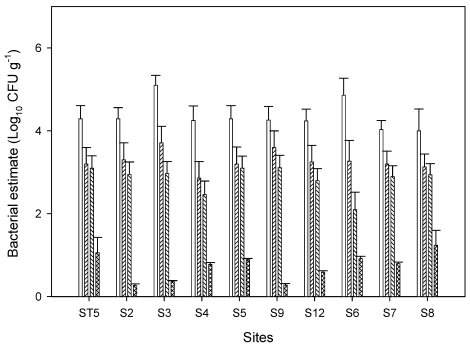
Concentrations of indicator bacteria in sediment on various sampling points along the major sources. Samples ST5, S2, S3, S4, S5, S9, and S12 are from urban runoff and samples S6, S7, and S8 are from agricultural inputs. Symbols □ total coliform (TC), ▨ fecal coliform (FC), ▧ *E. coli*, ▩ pathogenic *E. coli*. Error bars represent standard errors of three replicate samples. Only samples with potential pathogenic *E. coli* are shown.

Indicator bacteria in sediment and surface water were determined on 447 water and sediment samples collected from 19 sites over a 12-month period. Counts ranged from undetectable (detection limit 1 MPN 100mL**^−^**^1^) in the surface water to 2.5 × 10^4^ MPN 100g**^−^**^1^ in the sediment. Basic univariate summary statistics for TC, FC, *E. coli* and pathogenic *E. coli* counts included in Figure 2 are from sediment samples because most of the potentially pathogenic *E. coli* were recovered from sediment samples. The statistics summarized the log_10_ transformed counts for each indicator bacterial group. Total coliform counts were the highest, and with the greatest variability in concentrations. Presumptive pathogenic *E. coli* were small in numbers and most of the times below 10 cfu/g. 

### 3.2. Characterization Pathogenic *E. coli*

A total of about 389 isolates from the selected sites in Figure 2 were screened on CT-SMAC agar and these isolates were rescreened by PCR for various virulence factors. Only 17 isolates showed the presence of a combination of these factors (Table 2). These isolates were sent to Pennsylvania State University *E. coli* Reference Center for complete typing (Table 2). All strains were classified as non-O157. Two isolates were classified as *E. coli* O11:H40 and in 15 the O serotype could not be determined, but were identified as H11. 

Seven antibiotics were used for susceptibility tests of the 17 isolates. Resistant phenotypes were determined for the 17 isolates from CT-SMAC plates. All isolates were resistant to rifampacin except S12, followed by erythromycin and ampicillin. Only isolates from ST5 sites showed multiples resistances to rifampacin, erythromycin, and ampicillin. Site S8 also showed multiple resistances to ampicillin, rifampicin, and tetracycline. Site S8 is located along Cypress channel and this site is highly impacted by agricultural (dairy) activities. 

**Table 2:** Antimicrobial susceptibility, genetic variations, and resistant genotypes of selected isolates of presumptive pathogenic *E. coli*. *

Sample Name	Amox	Amp	Ceph	Eryth	Rif	Strep	Tet	LT	STa	STb	CNF1	CNF2	O	H	stx2	stx1	eae	*bla*_TEM_	*aadA*I	*tetA*	*tetB*	*tetC*
S8-1	S	S	S	I	R	S	S	-	-	-	-	-	11	40	-	-	-	-	-	-	-	-
S8-2	S	S	S	I	R	S	S	-	-	-	-	-	11	40	-	-	-	-	-	-	-	-
S8-3	S	R	I	I	R	S	R	-	-	-	-	-	-	11	-	-	+	-	-	-	-	-
S8-4	S	S	S	I	R	S	S	-	-	-	-	-	-	11	-	-	+	-	-	-	-	-
S8-5	S	S	S	I	R	S	R	-	-	-	-	-	-	11	-	-	+	-	-	-	-	-
S8-6	S	S	S	R	R	S	S	-	-	-	-	-	-	11	-	-	+	-	-	-	-	+
ST5-1	S	R	S	R	R	S	S	-	-	-	-	-	-	11	-	-	+	-	-	-	-	+
ST5-2	S	R	S	R	R	S	S	-	-	-	-	-	-	11	-	-	+	-	-	-	-	+
ST5-3	S	S	R	R	R	S	S	-	-	-	-	-	-	11	-	-	+	-	-	-	-	-
ST5-4	S	R	S	R	R	S	S	-	-	-	-	-	-	11	-	-	+	-	-	-	-	+
ST5-5	S	R	S	R	R	S	S	-	-	-	-	-	-	11	-	-	+	-	-	-	-	+
ST 5-6	S	S	S	R	R	S	S	-	-	-	-	-		11	-	-	+	-	-	-	-	+
ST5-7	S	S	S	R	R	S	S	-	-	-	-	-	-	11	-	-	+	-	-	-	-	+
ST5-8	S	S	S	R	R	S	S	-	-	-	-	-	-	11	-	-	+	-	-	+	-	+
ST 5-9	S	S	S	R	R	S	R	-	-	-	-	-	-	11	-	-	+	-	-	-	-	+
ST5-10	S	S	S	I	R	S	S	-	-	-	-	-	-	11	-	-	+	-	-	-	-	-
S12	S	R	R	I	I	I	S	-	-	-	-	-	-	11	-	-	+	-	-	-	-	-

* S = antimicrobial susceptibility, R = resistance, I = intermediate; - undetectable and + detectable genes by PCR. The six different isolates from site S8 are sediment samples from April 2005 and the ST5 from three sampling days.

Sites ST5 receives water from mostly urban sources from the watershed before it flows through the Prado wetland. One isolate from S8 and seven from ST5 were resistant to *tet*C genes based on positive PCR signal. Genes for ampicillin resistance (*bla*_TEM_) and streptomycin resistance (*ant3*"*)-Ia* (also called *aadA1*) were not detected in any of the samples that showed resistant phenotype, however, *tet*C gene was detected in most of the samples that were susceptible to tetracycline. 

### 3.3. Genetic Variations among *E. coli* Isolates from Watershed

**Figure 3:**
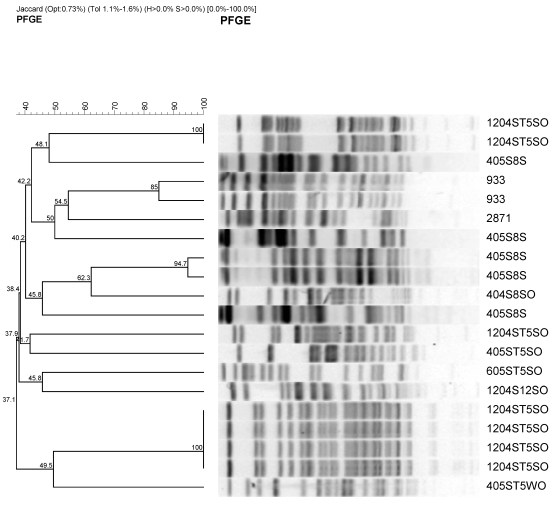
Representative PFGE fragment patterns and dendrogram analysis of potentially pathogenic antimicrobial-resistant *E. coli* stains from urban watershed impacted by different sources of pollutants. Most of the *E. coli* isolates were obtained from sediment except one that was obtained from surface water (405ST5 WO). Sample identifications are as follow; e.g., 405S8S and 1204ST5SO indicates month (4 or 12) followed by year (05 or 04), site description (S8 (1 to 6) or ST5-1 to 10 from Table 2), and sample source SO or S (sediment) or water (WO). 933 and 2,871 are pathogenic and nonpathogenic *E. coli* controls.

The 17 isolates were characterized by pulsed field gel electrophoresis using XbaI restriction endonucleases (Figure 3). 

Most of the restriction endonuclease digestion profiles clustered within specific sources. Most of the isolates from S8 site along Cypress channel clustered together with one exception. This isolates were collected on April 2004. This site is mainly agricultural source. The other cluster was isolates from urban runoff that flows through Prado wetland. This shows that *E. coli* isolates from the different sources have different PFGE profiles. 

### 3.4. Fecal Indicator Bacterial Levels in Source Water and Aquifer Sand Material During Sand Filtration

**Figure 4:**
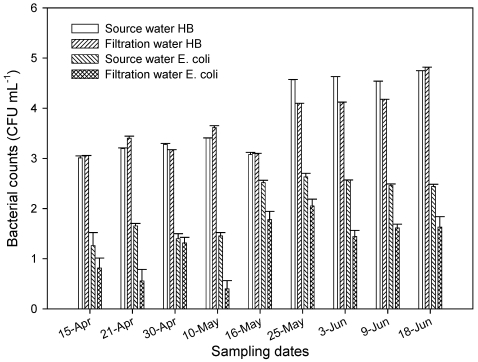
Levels of heterotrophic bacteria (HB) and *E. coli* as determined by plate count in the source water and after filtration through aquifer sand material. All samples were taken in April through June 2004.

There were no differences in the levels of heterotrophic bacteria as determined by plate count in the source water (influent) and the filtration (water that has gone through sand filtration (Figure 4). Significantly higher levels (P < 0.001) of HPC were found in water samples in late May and June than in April and early May. There were significant (*P* = 0.05) higher numbers of *E. coli* in source water in May and early June than in April. After water has gone through filtration tanks containing aquifer sand material, there was a 1 to 2 log reduction in *E. coli* in aquifer sand tank. This showed that the filtration unit with aquifer material had limited impact on *E. coli* population. 

## 4. Discussion

Examination of each site throughout the watershed indicated that indicator bacterial concentrations along Chino Creek and Cypress channel routinely exceeded the applicable water quality objectives. The exception was TC in the control sites (S1, M1) and WWTPs. The same trends were observed for fecal coliform [23]. Therefore, Figure 2 presents data mainly from the sediment of these two channels (Chino and Cypress) to illustrate the major sources of pollution to the watershed. From our previous studies, peak concentrations of *E. coli* depended more on larger storms and on pervious-area bacteria sources and loading rates [24]. Only the larger storms generated runoff, and thus bacteria wash off, on areas in the Chino Basin. Land uses that were assigned the highest bacteria-loading (Chino Creek and Cypress channel) values affected the time-averaged bacteria loads and the frequency of concentrations exceeding 235 cfu/100mL [24]. Therefore land use was the major factor affecting the concentration of *E. coli* in the Chino Basin area of the watershed. In contrast, Prado Park and open space land use areas had a significant decrease in the frequency that bac­teria concentrations in waterways exceeded 235 cfu/100mL. 

The continuous and increasing use of antibiotics has led to the emergence of pathogenic bacteria that are resistant to many antibiotics [25]. In this study, more that 50% of our isolates were resistant to *tet*C gene and three isolates carry resistant phenotype. Since tetracycline resistance genes are located on the mobile genetic elements, they are transmissible between bacteria. Two of these isolates are from agricultural sources and one is from urban source. Also, eight isolates from urban sources carry *tet*C resistance gene while one from agricultural sources carried this resistant gene. Therefore, most fecal bacteria from human or agricultural sources released into the environment may carry antibiotic resistance genes [26]. Their fate and the transfer of antibiotic resistances by gene transfer to other bacteria are of great concern to human health [27]. The great threat to drinking water of antibiotic resistant bacteria may be the high concentrations of such bacteria in the source water that may result in the transfer of genetic elements from nonpathogenic to pathogenic strains. This was confirmed by our recent study with 600 isolates of generic *E. coli* from the same watershed [9]. Resistance genes are often associated with integrons or mobile DNA elements such as plasmids and transposons that facilitate the spread of resistance genes [28,29,30]. More often, there is a linkage between many of these resistance genes on mobile elements and the distribution of antibiotic resistant bacteria in the environment [17,18,31]. We did not study the exact mechanisms of resistance in the current work; however previous molecular studies have shown strong statistical associations between resistance genes [32,33]. No isolate showed resistance to amoxicillin-clavulanic acid and this was in agreement with our previous study using 600 generic *E. coli* [9] that showed less that 2% were resistant to this antibiotic from urban runoff and none from agricultural sources. The correlation between antimicrobial resistance and the presence of antibiotic resistant genes was better for Streptomycin than than tetracycline and ampicillin. *aad*AI gene for streptomycin was not detected in any of our samples and this corresponded to all our isolates being susceptible to streptomycin (Table 2). However, such a correlation was not observed with ampicillin and tetracycline.

Pathogens with increased resistances may be transported from the animal or human via feces or other mechanism into rivers and groundwater [11,34] where the water is use as a source for domestic water supply. In this watershed, there are networks of channels and creeks that surface water are transported to the Santa Ana River. Through this process antibiotic resistant bacteria may be transported from human or animal sources to the river that is subsequently used to recharge ground water for domestic water use. In a study to determine the impact of nontherapeutic use of antibiotics on swine manure-impacted water sources, surface water and groundwater situated up and down gradient from a swine facility were assessed for antibiotic-resistant enterococci and other fecal indicators. As expected, the median concentrations of enterococci, fecal coliforms, and *Escherichia coli* were 4 to 33 fold higher in down-gradient *versus* up-gradient surface water and groundwater [5]. Higher amounts of erythromycin- and tetracycline-resistant enterococci were detected in down-gradient surface waters. These findings demonstrated that water contaminated with swine manure could contribute to the spread of antibiotic resistance in the environment. Recently, Ibekwe *et al*. [9] found in the watershed used for this study that more *E. coli* with higher multiple resistant phenotypes were present in water samples from urban sources that from agriculture sources. These isolates were also more diverse genetically that isolates from agricultural sources. Therefore, there is no doubt whether there are fecal bacterial is drinking water sources, but the question is how we manage such systems that these bacteria are not in the actual drinking water. This is achievable in developed countries but this is a serious problem in developing countries because of inadequate water treatment plants. 

The microbiological data provided in this study can help water utility companies in their understanding of source water quality and help them in the processing of tertiary treated water that may be subsequently available for domestic use. After water has gone through the filtration tanks containing aquifer material, there were reductions in *E. coli* population. The data presented here demonstrates high level of indicator bacteria in the river as it continues to the ocean. Our study also suggest that when surface water is diverted through aquifer sand material significant reduction in fecal bacterial population occurs as the water passes through aquifer sand material by a natural filtration process. Part of the source water (Santa Ana River) used for this study has gone through tertiary treatment and wetlands before flowing into the artificial lakes at the Orange County Water District field station. Water from these lakes is subsequently used for ground water recharge and discharge. After this process the water is treated further for domestic use by over 2 million residence and businesses. Part of the Santa Ana River continues to flow and empties into the Pacific Ocean near Huntington Beach. In southern California, it is well recognized that a major cause of bacterial pollution of coastal waters is urban runoff in rivers/channels and storm drains that discharge into the ocean [35,36]. In a recent paper enumerating enterococci in marine and intertidal sediments [37], high densities of fecal indicator bacteria were reported in Santa Ana River near Huntington Beach. These authors indicated that shoreline waters at Huntington State Beach may be recipients of fecal indicator bacteria originating from intertidal sediments in the Santa Ana River that contain high levels of bacteria.

## 5. Conclusions

In this study no *E. coli* O157 was found in the surface water or sediment despite the fact that the study area has one of the highest concentrations of cattle in the United States and cattle is the main reservoir of pathogenic *E. coli*. The concern is that the presence of antibiotic resistant non pathogenic *E. coli* in this watershed is a matter of great concern because of possible horizontal gene transfer from generic to pathogenic *E. coli* which may lead to increased duration and severity of morbidity. 
